# Cardiac resynchronization therapy improves heart failure in one patient with acromegaly-induced cardiomyopathy: a case report

**DOI:** 10.1186/s13256-019-2030-y

**Published:** 2019-04-25

**Authors:** Jun yi Wang, Yong mei Hu, Jian xiong Liu, Xiao jia Luo

**Affiliations:** Affiliated with Chengdu Second People’s Hospital, Qinyun South Road, 10th Avenue, Chengdu, 610041 Sichuan China

**Keywords:** Acromegalic cardiomyopathy, Heart failure, Cardiac resynchronization therapy

## Abstract

**Background:**

Congestive heart failure is rarely observed in patients with acromegaly. Excessive growth hormone secretion and elevation of insulin-like growth factor 1 contribute to pathological changes in myocyte growth and structure, cardiac contractility, vascular function, and in later stage may progress to cardiac dysfunction. Early recognition of the condition is paramount, though the insidious progression of the disease commonly results in late diagnosis. Current standard regimens of pharmacological therapy, surgical treatment, radiotherapy are designed to normalize serum levels of both insulin-like growth factor 1 and growth hormone. In patients with late-stage heart failure due to acromegalic cardiomyopathy, cardiac resynchronization therapy might be a desirable treatment to help cardiac synchronization, improve symptoms, and eventually reduce hospital admissions together with mortality rates.

**Case presentation:**

We describe a case of a 49-year-old man with a history of acromegaly who presented to our hospital with a diagnosis of decompensated systolic heart failure. Serial electrocardiograms showed wide (160–200 ms) QRS duration with left bundle branch block. Echocardiography showed severe left ventricular dysfunction that simultaneously achieved a left ventricular ejection fraction of 16%. Surgical indication was rarely assessed by neurosurgeons. Given that the stereotactic radiosurgery together with pharmacotherapy produced infinitesimal effects, cardiac resynchronization therapy was performed. Owing to biventricular synchronization and holding back reverse remodeling, the patient’s symptoms were successfully alleviated, and he was discharged from the hospital.

**Conclusions:**

Congestive heart failure is a rare complication in acromegaly-induced cardiomyopathy (occurs in only 3% of patients). Early diagnosis and treatment with curative drugs more than cardiovascular implantable electronic devices might lead to better surgical outcomes in this group of patients.

## Background

Acromegaly presents with multisystem involvement, and cardiac manifestations remain an important cause of mortality [1, 2]. Chronic excess of growth hormone (GH) and insulin-like growth factor 1 (IGF-1) leads to the development of acromegalic cardiomyopathy [3]. The clinical manifestations are biventricular hypertrophy, diastolic dysfunction, and in later stages may progress to systolic dysfunction and congestive heart failure [4]. Long-term treatments of acromegaly with pharmacological therapy, surgical treatment, and radiotherapy are extensively identified as standard regimens, among which surgical resection of the pituitary adenoma is often the first-line treatment [5]. In patients either waiting for or missing surgical indications due to severe heart failure, cardiac resynchronization therapy (CRT) might act as a desirable treatment to help synchronize cardiac contractility, improve symptoms, and reduce hospital admissions and mortality rates in patients with moderate to severe heart failure, hence ultimately creating chances for operation.

We report a case of a patient with acromegaly who was diagnosed with severe cardiac failure at the time of diagnosis and failed to recover cardiac function after pharmacotherapy and radiotherapy. Though successful resection of the pituitary adenoma was crucial, low left ventricular ejection fraction (LVEF) value in alignment with poor cardiac function could have made the surgery life-threatening. We applied mechanical therapy as CRT to this patient, which helped better control acromegalic cardiomyopathy, and this approach may create further chances for subsequent surgical resection.

## Case presentation

**Fig. 1:**
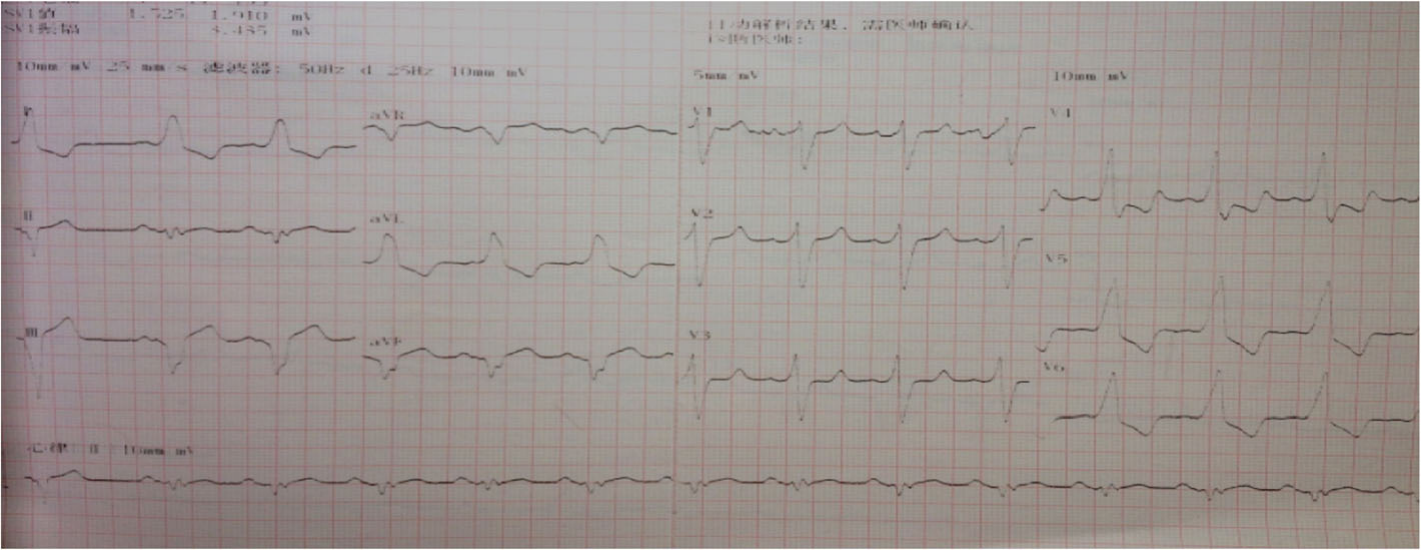
Electrocardiogram on admission demonstrating sinus rhythm with wide (160 ms) QRS duration of left bundle branch block

A 49-year-old man with a history of acromegaly was admitted to our hospital with the concern of recurrent shortness of breath and dyspnea on exertion during the previous 2 years, and he had experienced an episode of presyncope 2 weeks prior without any further evaluation. He was a chef in a local restaurant for almost 30 years. He had no family history of any diseases and no past history of hypertension, diabetes mellitus, sleep apnea, or sudden cardiac death. He did not smoke or consume alcohol. The patient provided a history of stereotactic radiosurgeries twice in a decade or so and adherence to treatment with a somatostatin analog (octreotide given 40 mg once per month through intramuscular injection) at the time of diagnosis 20 years before. The patient was overweight and moderately nourished. He was 1.85 m (73 inches) tall, weighed 134 kg, and had a body mass index of 39 kg/m^2^. His blood pressure was 110/60 mmHg, and his heart rate was 92 beats/min with sinus rhythm. He had distinct skeletal features that included prominent superciliary arches and nose bridge, enlargement of the tongue and lip, and large hands and feet. Cardiac auscultation revealed irregular premature beats and pathological third heart sound, and a systolic murmur was discovered over the apex and aortic area. Bilateral extensive borders of cardiac dullness were noted. His physiological reflexes were present without any pathology. An electrocardiogram demonstrated sinus rhythm with wide (160 ms) QRS duration of left bundle branch block (LBBB) (Fig. 1). The patient’s condition was classified as New York Heart Association (NYHA) stage III–IV.

On admission, magnetic resonance imaging showed pituitary macroadenoma. Given the symptoms described, we arranged blood testing of myocardial injury markers showing an elevated brain natriuretic peptide level of 740 pg/ml indicating cardiac failure (Table 1). Hormone laboratory tests performed subsequently demonstrated excessive secretion of GH and IGF-1, twofold greater than the reference normal upper limit, which was consistent with pituitary macroadenoma (Table 2). Other routine analyses of liver and renal function were roughly normal.

**Table 1:** Myocardial injury markers on admission

Measured value	Reference range
CK-MB mass < 1.0 ng/ml	0–4.3 ng/ml
Myoglobin 50.8 ng/ml	0–107 ng/ml
Troponin I 0.07 ng/ml	0–0.40 ng/ml
BNP 740 pg/ml	0–100 pg/ml
D-dimer 723 ng/ml	0–600 ng/ml

*BNP* Brain natriuretic peptide, *CK-MB* Creatine kinase MB

**Table 2:** Initial hormone laboratory tests

	Measured value	Reference range
GH	32.5 ng/ml	0–2.10 ng/ml
IGF-1	627.0 ng/ml	117.0–329.2 ng/ml
TSH	0.852 mIU/L	0.35–4.94 mIU/L
fT4	14.80 pmol/L	9.00–19.00 pmol/L
PRL	8.60 ng/ml	3.6–16.3 ng/ml

*Abbreviations: fT4* Free thyroxine, *GH* Growth hormone, *IGF-1* Insulin-like growth factor 1, *PRL* Prolactin, *TSH* Thyroid-stimulating hormone

**Fig. 2:**
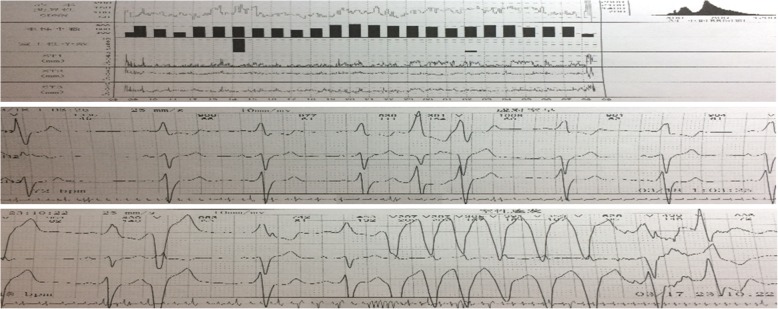
Dynamic electrocardiogram showing frequent ventricular premature beats and nonsustained ventricular tachycardia

A Holter monitor was ordered for underlying arrhythmias to explain the patient’s dyspnea, chest discomfort, and presyncope. It demonstrated sinus rhythm with an average heart rate of 68 beats/min, frequent ventricular premature beats, and nonsustained ventricular tachycardia (up to 2200 ms) (Fig. 2).

A chest x-ray showed a cardiothoracic ratio (CTR) of 78%. Echocardiography showed diffuse impairment of left ventricular (LV) systolic motion, reaching an LVEF of 16%. We noted hypertrophy of the ventricular septum at 18 mm, ventricular dilation, with LV diameter of 72 mm. The right ventricle and atrium and the left atrium were also dilated with moderate mitral regurgitation and mild tricuspid regurgitation. There was no associated systolic anterior motion (SAM) of the mitral valve. Dyssynchrony of the biventricular systolic motion was apparent.

Given an exertional component to the symptoms together with echo presentations in order to better exclude ischemic cardiomyopathy, coronary angiography was performed, which showed normal coronary arteries without stenosis, and left ventriculography applied simultaneously revealed an EF of 20% with diffuse LV hypokinesis.

Given the patient’s previous medical history of acromegaly, the absence of obstructive coronary artery imaging findings or segmental dyskinesia, family history of hypertrophic cardiomyopathy (HCM), symmetric hypertrophy, as well as absence of SAM of the mitral valve, acromegaly-induced cardiomyopathy was confirmed, which was absolutely opposed to coronary heart disease (CHD) and HCM.

These results indicated that it was probably not a case of hereditary cardiomyopathy; therefore, we diagnosed the patient as having secondary dilated cardiomyopathy due to acromegaly, even taking it a step further progressing to congestive heart failure secondary to acromegaly-induced dilated cardiomyopathy.

**Fig. 3:**
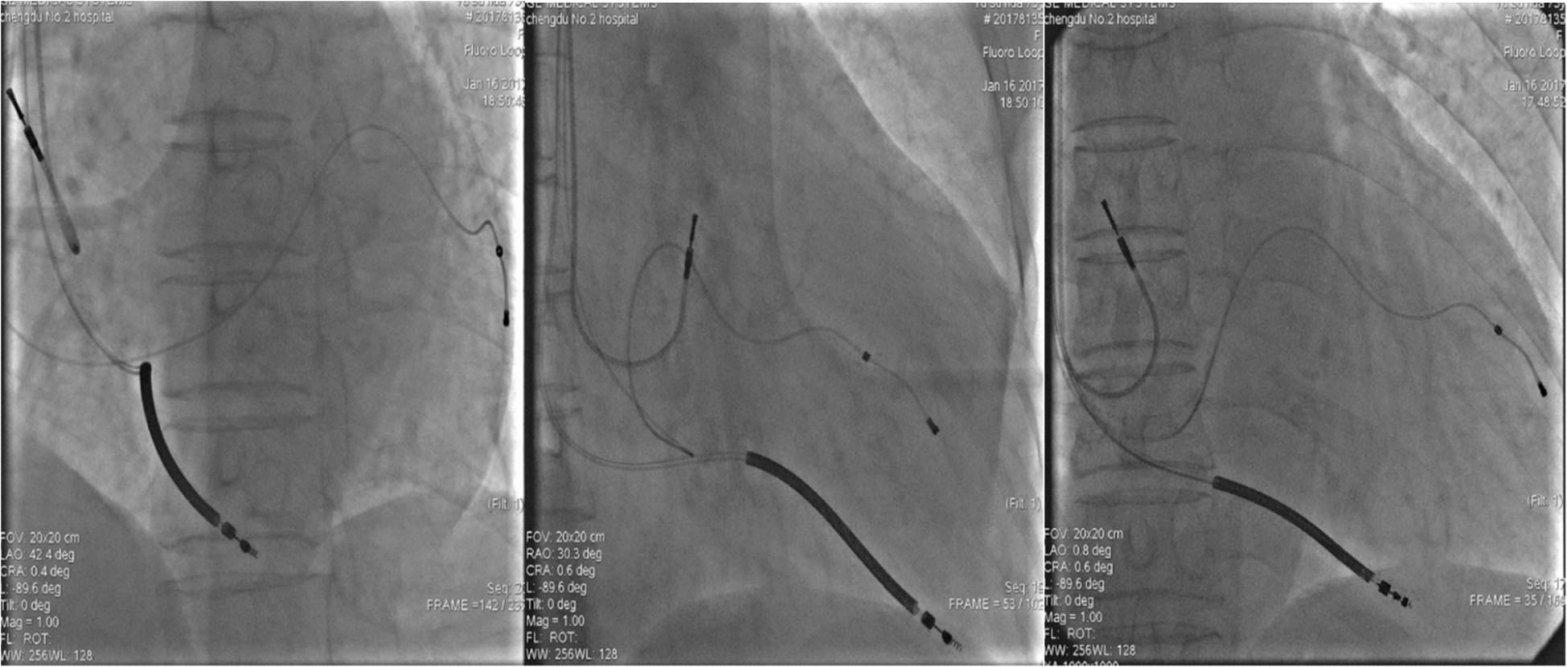
Cardiac resynchronization therapy electrode position in LAO 45 degrees, RAO 30 degrees, AP view. *LAO* Left anterior oblique, *RAO* Right anterior oblique, *AP* Anteroposterior position

Chronic excess of GH and IGF-I secretion affects cardiac morphology and performance [5], so etiological treatment for acromegaly-induced cardiomyopathy is crucial to suppressing GH secretion or blocking GH action for the sake of reversing acromegaly-induced cardiomyopathy. The mainstay of treatment acknowledged globally is surgical resection of the pituitary adenoma [6], which was unfortunately considered high-risk given our patient’s cardiac condition (NYHA stage III–IV). Although stereotactic radiosurgery combined with somatostatin analogs and GH antagonists administrated previously were effective in suppressing hormones, they could not help his cardiac function. Therefore, we carefully administered diuretics, vasodilators, angiotensin-converting enzyme inhibitor (ACEI), β-blockers, and spironolactone for management of heart failure following the current guidelines [7]; in the meantime, octreotide (200 μg/day) was administered for the control of GH excess. After good compliance of pharmacotherapy and a regular medical examination regimen for nearly half a year, the serum GH and IGF-1 concentrations decreased from 32.50 ng/ml to 1.98 ng/ml and 627.00 ng/ml to 229.10 ng/ml, respectively, but the patient was hospitalized again because of uncontrollable cardiac failure. Accompanied by the normalization of GH and IGF-1 levels, the patient’s cardiac function did not seem to take a favorable turn upon readmission. Though echocardiography showed a recovered EF value from 16% to 28%, a significant ventricular mechanical dyssynchrony was detected as formerly. Electrophysiological study was performed using a nonaggressive stimulation protocol, which revealed a nonsustained ventricular monomorphic tachycardia [8]. In the presence of overt ventricular dyssynchrony, complete LBBB, LVEF< 35%, inducible ventricular tachycardia, and symptomatic heart failure despite guideline-directed medical therapy, surgical indication was rarely assessed by neurosurgeons, and stereotactic radiosurgery together with pharmacotherapy produced infinitesimal effects. Therefore, we boldly recommended cardiac resynchronization therapy with defibrillator (CRT-D) implantation based on device implantation official guidelines [7, 9]. The patient underwent CRT insertion finally and was discharged to home 5 days later, pharmacotherapy continued as usual (Fig. 3).

**Fig. 4:**
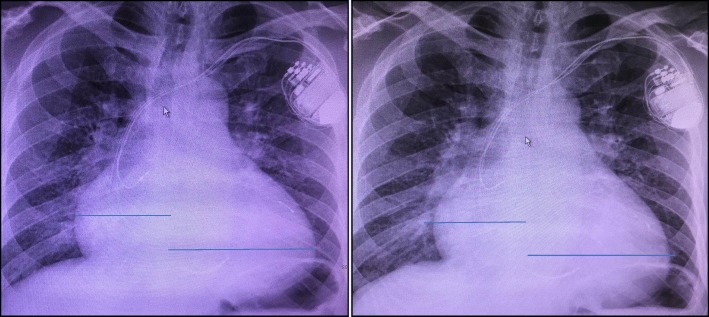
Chest x-rays at 1 month (left) and 6 months (right) after cardiac resynchronization therapy implantation, during follow-up visits

Telephone follow-up was arranged, and the patient claimed symptom improvement following the device insertion 1 month later and was basically back to normal life. We required that he return for follow-up at 1 month, 3 months, and 6 months after the interventional therapy. The patient has been followed in our outpatient clinic for nearly half a year now. During his last visit, echocardiography identified improved LVEF of 54%, and a chest x-ray showed reduced CTR of 60%. The patient was in NYHA functional class II (Fig. 4).

## Discussion and conclusions

We present a case of a patient with acromegaly who had clinical signs and symptoms of refractory decompensated heart failure, diagnosed as congestive heart failure (CHF) and attributed to acromegaly-induced dilated cardiomyopathy. He eventually had a dramatic response to medical device therapy. This case reinforces the importance of careful examination, accurate diagnosis, detailed evaluation, and searching for better alternative treatments when standard therapy initially fails.

Acromegaly is a rare endocrine disorder with an incidence of 3 per 1 million per year [1]. Acromegaly is characterized by chronic GH and IGF-1 hypersecretion that leads to an increased mortality rate, with cardiovascular complications accounting for the highest number of patient deaths [2, 4, 6]. GH exerts its effects by stimulating the production of IGF-I either directly or indirectly, which mediates GH action on peripheral tissues. The GH/IGF-1 axis acts through increasing biological protein synthesis, cardiomyocyte size, and muscle-specific gene expression. Specifically, IGF-1 promotes cardiac hypertrophy and increases muscle-specific gene transcription (namely, troponin I, myosin light chain 2, and α-actin) [10, 11]. Chronic excess of these hormones leads to the development of acromegalic cardiomyopathy, which in early stages manifests biventricular hypertrophy, diastolic dysfunction, and later may progress to systolic dysfunction and eventually congestive heart failure [3]. Heart failure from acromegalic cardiomyopathy is one of the most common causes of death in acromegaly and is now receiving increasing attention. The GH/IGF-1 axis regulates cardiac growth, stimulates myocardial contractility, and influences the vascular system. Epidemiological evidence suggests that serum IGF-1 levels in the low-normal range are associated with increased risk of acute myocardial infarction, ischemic heart disease, coronary and carotid artery atherosclerosis, and stroke [12–14]. Among histological abnormalities induced by the hormones, the most relevant is a proliferation of myocardial fibrous tissue leading to progressive interstitial remodeling and deterioration of diastolic and systolic cardiac performance [15]. In acromegaly, chronic GH/IGF-I excess causes a concentric cardiac hypertrophy associated with diastolic dysfunction. In later stages, impaired systolic function ending in heart failure may occur. Abnormalities of cardiac rhythm (such as premature beats, paroxysmal atrial fibrillation, paroxysmal supraventricular tachycardia, sick sinus syndrome, ventricular tachycardia, and bundle branch blocks) and of cardiac valves (the most frequent being mitral and aortic abnormalities associated with LV hypertrophy) can also occur [15–17]. The yearly incidence of acromegaly is estimated to be between 3 and 4 cases per 1 million people per year. The average age at diagnosis is 40 to 45 years [18, 19], and our 49-year-old patient was nearly in that age range.

Current treatments aim to normalize GH and IGF-1 secretion levels in patients with acromegaly for the sake of reversing some of the morphological changes in the early stage, regression of cardiac hypertrophy and improvement of cardiac dysfunction [4, 18–20]. Different strategies for management can be divided into two components, namely to control GH and IGF-1 hypersecretion and cardiac heart failure. The normalization of GH and IGF-1 levels, whether by surgical or pharmacological therapy, is essential to reversing or arresting cardiovascular complications [5]. An earlier case report described a 43-year-old man with severe congestive heart failure produced by acromegalic cardiomyopathy who recovered significantly through octreotide followed by transsphenoidal surgery [21]. Some reports suggest that the beneficial effects obtained from hormone suppression therapy appear more significant in younger patients with short disease duration than in older patients [22]. Other reports describe how the classic treatments of acromegaly, including surgical resection of the pituitary adenoma, somatostatin analogs and GH antagonists, and stereotactic radiosurgery or fractionated radiation, might improve cardiac function in the short term but probably have very little effect on long-term prognosis [23, 24]. Successful later case reports support the idea of the use of traditional heart failure therapy, which consists of ACEIs, angiotensin receptor blockers, β-blockers, aldosterone antagonists, and diuretics, to further improve cardiovascular function because GH/IGF-1 control alone is insufficient [25, 26]. Heart transplant is an option for end-stage heart failure [24, 27]. Considering our patient’s minimal response to these acknowledged treatments mentioned above, we carefully selected CRT in this patient, and his cardiac function recovered considerably with our elaborate care. At his follow-up visits, the patient has been in good condition, with various gradually improved cardiac function indexes.

CRT is an effective and well-established therapy for patients with heart failure, LV systolic dysfunction (LVEF ≤35%), and electrical dyssynchrony, demonstrated by a surface QRS duration of ≥ 130 ms [7]. It appears that CRT offers a more favorable response with regard to reverse LV remodeling in cases of nonischemic cardiomyopathy [28, 29]. The use of mechanical therapies including CRT/CRT-D implantation have not been adequately studied in acromegalic heart failure, and the long-term prognosis of CHF in patients with acromegaly remains poor. This group of patients might benefit from device therapies that restore ventricular systolic synchrony and ameliorate ventricular reverse remodeling.

## Abbreviations

ACEI: Angiotensin-converting enzyme inhibitor
BNP: Brain natriuretic peptide
CHF: Congestive heart failure
CRT: Cardiac resynchronization therapy
CRT-D: Cardiac resynchronization therapy with defibrillator
CTR: Cardiothoracic ratio
GH: Growth hormone
HCM: Hypertrophic cardiomyopathy
IGF-1: Insulin-like growth factor 1
LBBB: Left bundle branch block
LV: Left ventricular
LVEF: Left ventricular ejection fraction
MR: Mitral regurgitation
NYHA: New York Heart Association
SAM: Systolic anterior motion
